# Enteropathogenic *Escherichia coli* (EPEC) Causes Chronic Diarrhea and Hyponatremia in an Adult

**DOI:** 10.1002/ccr3.70288

**Published:** 2025-03-27

**Authors:** Saisai Chen, Colin Feuille

**Affiliations:** ^1^ Department of Medicine University of California San Francisco San Francisco California USA; ^2^ Division of Gastroenterology, Department of Medicine University of California San Francisco San Francisco California USA

**Keywords:** chronic diarrhea, Enteropathogenic *E. coli* (EPEC), hyponatremia, infectious gastroenteritis

## Abstract

Enteropathogenic *Escherichia coli* (EPEC) is frequently identified on gastrointestinal pathogen multiplex polymerase chain reaction testing. However, it is unclear whether EPEC causes diarrhea in adults. We describe the case of a 48‐year‐old man living in the United States who presented with 6 weeks of large‐volume watery diarrhea and significant hyponatremia refractory to hypertonic saline and fluid restriction, who was found to have EPEC. Ciprofloxacin was initiated, resulting in the resolution of both diarrhea and hyponatremia. This case demonstrates that EPEC can be pathogenic in adults and can be associated with significant hyponatremia.

**Summary**

- EPEC is thought to cause severe diarrhea only among infants in developing countries.
- Although commonly identified by multiplex PCR, its pathogenicity in adults is unclear.
- In this case report, we identify EPEC as the most likely cause of chronic diarrhea and hyponatremia in an adult living in a developed nation.

## 1 Introduction

In the United States, there are an estimated 179 million cases of acute infectious diarrhea annually, resulting in over 200,000 hospitalizations and over 2500 deaths [1]. With the advent of multiplex polymerase chain reaction (PCR) technology, it has become easier than ever to screen stool samples for potential pathogens. For example, the BioFire Film Array Gastrointestinal Panel (GIP) (BioFire Diagnostics, Salt Lake City, UT) offers a multiplex PCR assay that screens diarrheal samples for 22 enteric pathogens [2]. BioFire GIP was approved in the United States in 2014 and is now used in many clinical microbiology labs. One of the most frequently identified enteric pathogens is Enteropathogenic *Escherichia coli* (EPEC). In a multicenter evaluation of the BioFire GIP array in the United States, EPEC was identified in 22% of samples across all age groups [2]. Although it is one of the leading causes of diarrhea‐related infant deaths in developing countries [3], there is controversy regarding EPEC pathogenicity in adults. For instance, in a study of hospitalized patients in Norway, among those in which EPEC was the only pathogen detected by multiplex PCR, 20% had no diarrhea and 73% had a clinical diagnosis of some other non‐infectious cause of diarrhea [4]. These observations have led some to disregard EPEC‐positive PCR results. In this case report, we discuss a 48‐year‐old man who presented with chronic watery diarrhea and severe hyponatremia found to be EPEC positive on the BioFire GIP.

## 2 Case Presentation

A 48‐year‐old man with alcohol use disorder in remission presented to the emergency room of a county hospital for an evaluation of 6 weeks of postprandial watery diarrhea. He has a history of housing insecurity, and his symptoms were interfering with activities of daily living. He reported 10–15 episodes of large‐volume watery diarrhea each day, without fever, vomiting, or abdominal pain. He had no recent travel. On admission, he was afebrile, tachycardic, and hypertensive. His blood glucose was 455 mg/dL, and hemoglobin A1c was 10.9%. He was also hyponatremic to 127 mEq/L (corrected to 133 mEq/L).

## 3 Investigations and Treatment

The patient's persistent diarrhea was initially thought to be exocrine pancreatic insufficiency due to chronic pancreatitis secondary to prior long‐term alcohol use. However, fecal fat was normal, fecal elastase was found to be within normal limits, and magnetic resonance cholangiopancreatography showed no evidence of chronic pancreatitis. The absence of blood or mucus in the stool and a normal fecal calprotectin argued against inflammatory diarrhea. Infectious diarrhea was initially deemed less likely given the time course of his diarrhea, absence of fevers and abdominal pain, and normal WBC count. HIV testing was negative. Toxigenic *Clostridioides difficile* PCR was negative. Stool ova and parasite analysis was also negative. A BioFire GIP resulted positive for EPEC only. The patient was treated with ciprofloxacin, and after the initiation of antibiotics, stool frequency decreased, and stool consistency returned to normal.

His hyponatremia was treated with hypertonic saline, sodium tablets, and fluid restriction of 1.5 L per day. However, his sodium decreased to 122 mEq/L even as blood glucose levels normalized after insulin administration. Further testing revealed urine sodium 163 mEq/L and urine osmolality 420 mOsm/kg, consistent with the syndrome of inappropriate anti‐diuretic hormone (SIADH). Evaluation for alternative causes of SIADH, including chest x‐ray to screen for lung masses, did not reveal another explanation. Surprisingly, the patient's hyponatremia began to resolve spontaneously following the initiation of ciprofloxacin for EPEC treatment.

## 4 Discussion

EPEC is a significant cause of infant diarrhea in developing countries and is associated with high mortality rates [3]. It is also one of the most frequently detected pathogens on GI pathogen multiplex PCR testing [2]. Since patients are often asymptomatic or have co‐infection with another pathogen, it has been questioned whether EPEC should be treated at all. We report a case in which EPEC caused significant and persistent watery diarrhea in an immunocompetent 48‐year‐old man living in the United States. While previous reports suggest that stool frequency in EPEC‐positive individuals averages 4–5 per day and symptom duration averages 11 days for those aged 41–64 years [5], our patient had 10–15 episodes of diarrhea per day lasting 6 weeks. The severity and duration of diarrhea caused by EPEC may be related to the specific serotype of EPEC as well as inoculum size and incubation time [6, 7]. Moreover, EPEC may cause disruption of intestinal microvilli and lead to malabsorption, which can cause nutritional deficiencies and persistent diarrhea [8].

In addition to the severity and duration of his symptoms, another intriguing aspect of our case is the significant hyponatremia. Little is known about the effect of EPEC infection on electrolyte levels in patients, especially adults. In one retrospective case–control study, the average serum sodium level of EPEC‐positive pediatric patients was significantly lower than that of EPEC‐negative controls [5]. However, among adults in the same study, serum sodium levels were not significantly different between EPEC‐positive and EPEC‐negative individuals, regardless of symptoms [5]. Interestingly, EPEC has been shown to alter sodium and chloride absorption in cell culture models [9]. In our case, the patient presented with severe hyponatremia with a nadir of 122 mEq/L, without symptoms attributable to hyponatremia. Serum and urine osmolality and urine sodium studies were consistent with SIADH. However, no evidence of malignancy or any alternative underlying etiology was found on imaging. Most importantly, upon initiating ciprofloxacin for the treatment of EPEC, his hyponatremia corrected spontaneously along with the resolution of his diarrhea. This suggests that EPEC infection was the cause of his hyponatremia.

## 5 Conclusion

In summary, we present a case of pathogenic EPEC infection causing diarrhea and SIADH resulting in severe hyponatremia. While many cases of EPEC may be asymptomatic in adults, this case argues for consideration of EPEC as a diarrheal pathogen, particularly when other causes of diarrhea have been excluded.

## Author Contributions

**Saisai Chen:** conceptualization, data curation, formal analysis, writing – original draft, writing – review and editing. **Colin Feuille:** data curation, formal analysis, supervision, writing – review and editing.

## Consent

Informed written consent was obtained for this case report.

## Conflicts of Interest

The authors declare no conflicts of interest.

## Data Availability

The data that support the findings of this study are available from the corresponding author upon reasonable request.
